# Supplementary figures and images for: Deciphering the pathogenicity of three NKX2-1 variants in ultra-severe forms of childhood interstitial lung disease
Source: PLoS One. 2025 Dec 19;20(12):e0338446. doi: 10.1371/journal.pone.0338446 (PMC12716760; doi:10.1371/journal.pone.0338446)

## Raw Western blot N=1

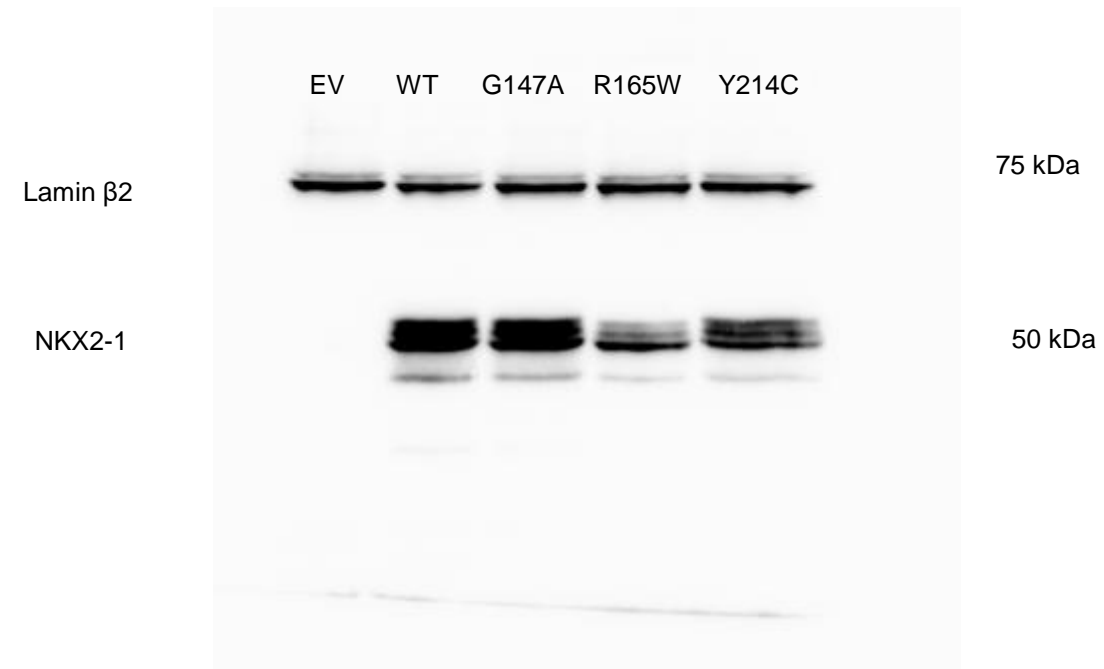

## Raw Western blot N=2

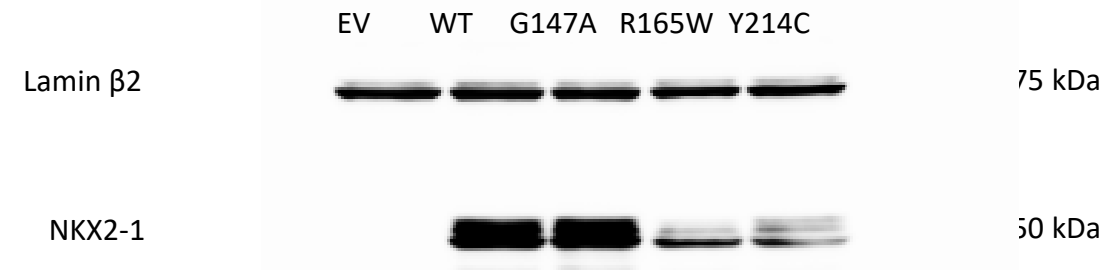

## Raw Western blot N=3

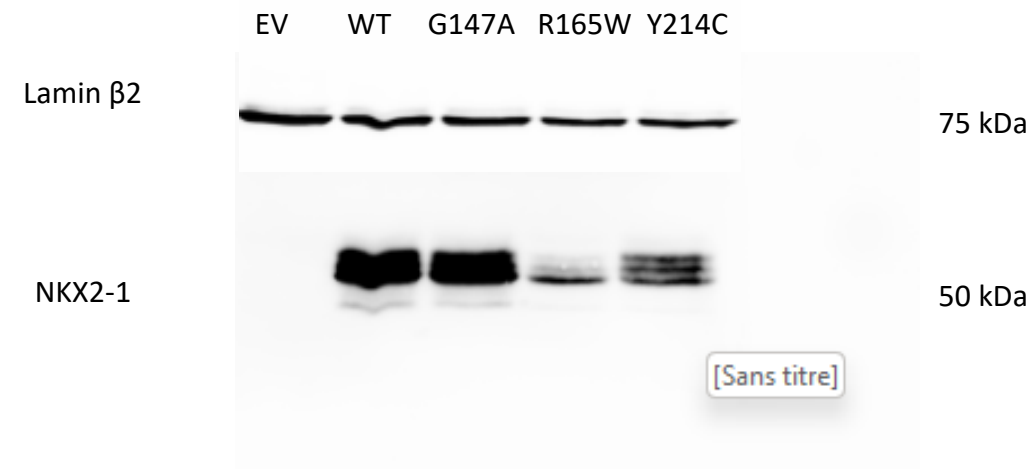

Supplement: S5 Fig — (PDF) [file pone.0338446.s005.pdf]
