# Supplementary material for: Deciphering the pathogenicity of three NKX2-1 variants in ultra-severe forms of childhood interstitial lung disease
Source: PLoS One. 2025 Dec 19;20(12):e0338446. doi: 10.1371/journal.pone.0338446 (PMC12716760; doi:10.1371/journal.pone.0338446)
Supplement: S1 File — (RTF) [file pone.0338446.s006.rtf]

NM_001079668    NM_003317       Patient 1 c.731A>G p.(Tyr244Cys)/  c.641A>G p.(Tyr214Cys)  ATGTGGTCCGGAGGCAGTGGGAAGGCGCGGGGCTGGGAGGCCGCGGCGGGAGGGAGGAGCAGCCCCGGCAGGCTCAGCCGCCGCCGAATCATGTCGATGAGTCCAAAGCACACGACTCCGTTCTCAGTGTCTGACATCTTGAGTCCCCTGGAGGAAAGCTACAAGAAAGTGGGCATGGAGGGCGGCGGCCTCGGGGCTCCGCTGGCGGCGTACAGGCAGGGCCAGGCGGCACCGCCAACAGCGGCCATGCAGCAGCACGCCGTGGGGCACCACGGCGCCGTCACCGCCGCCTACCACATGACGGCGGCGGGGGTGCCCCAGCTCTCGCACTCCGCCGTGGGGGGCTACTGCAACGGCAACCTGGGCAACATGAGCGAGCTGCCGCCGTACCAGGACACCATGAGGAACAGCGCCTCTGGCCCCGGATGGTACGGCGCCAACCCAGACCCGCGCTTCCCCGCCATCTCCCGCTTCATGGGCCCGGCGAGCGGCATGAACATGAGCGGCATGGGCGGCCTGGGCTCGCTGGGGGACGTGAGCAAGAACATGGCCCCGCTGCCAAGCGCGCCGCGCAGGAAGCGCCGGGTGCTCTTCTCGCAGGCGCAGGTGTACGAGCTGGAGCGACGCTTCAAGCAACAGAAGTACCTGTCGGCGCCGGAGCGCGAGCACCTGGCCAGCATGATCCACCTGACGCCCACGCAGGTCAAGATCTGGTTCCAGAACCACCGCTACAAAATGAAGCGCCAGGCCAAGGACAAGGCGGCGCAGCAGCAACTGCAGCAGGACAGCGGCGGCGGCGGGGGCGGCGGGGGCACCGGGTGCCCGCAGCAGCAACAGGCTCAGCAGCAGTCGCCGCGACGCGTGGCGGTGCCGGTCCTGGTGAAAGACGGCAAACCGTGCCAGGCGGGTGCCCCCGCGCCGGGCGCCGCCAGCCTACAAGGCCACGCGCAGCAGCAGGCGCAGCACCAGGCGCAGGCCGCGCAGGCGGCGGCAGCGGCCATCTCCGTGGGCAGCGGTGGCGCCGGCCTTGGCGCACACCCGGGCCACCAGCCAGGCAGCGCAGGCCAGTCTCCGGACCTGGCGCACCACGCCGCCAGCCCCGCGGCGCTGCAGGGCCAGGTATCCAGCCTGTCCCACCTGAACTCCTCGGGCTCGGACTACGGCACCATGTCCTGCTCCACCTTGCTATACGGTCGGACCTGGTGA                                                      Patient 2 c.583C>T p.(Arg195Trp)/ c.493C>T p.(Arg165Trp)ATGTGGTCCGGAGGCAGTGGGAAGGCGCGGGGCTGGGAGGCCGCGGCGGGAGGGAGGAGCAGCCCCGGCAGGCTCAGCCGCCGCCGAATCATGTCGATGAGTCCAAAGCACACGACTCCGTTCTCAGTGTCTGACATCTTGAGTCCCCTGGAGGAAAGCTACAAGAAAGTGGGCATGGAGGGCGGCGGCCTCGGGGCTCCGCTGGCGGCGTACAGGCAGGGCCAGGCGGCACCGCCAACAGCGGCCATGCAGCAGCACGCCGTGGGGCACCACGGCGCCGTCACCGCCGCCTACCACATGACGGCGGCGGGGGTGCCCCAGCTCTCGCACTCCGCCGTGGGGGGCTACTGCAACGGCAACCTGGGCAACATGAGCGAGCTGCCGCCGTACCAGGACACCATGAGGAACAGCGCCTCTGGCCCCGGATGGTACGGCGCCAACCCAGACCCGCGCTTCCCCGCCATCTCCCGCTTCATGGGCCCGGCGAGCGGCATGAACATGAGCGGCATGGGCGGCCTGGGCTCGCTGGGGGACGTGAGCAAGAACATGGCCCCGCTGCCAAGCGCGCCGCGCAGGAAGCGCCGGGTGCTCTTCTCGCAGGCGCAGGTGTACGAGCTGGAGCGACGCTTCAAGCAACAGAAGTACCTGTCGGCGCCGGAGCGCGAGCACCTGGCCAGCATGATCCACCTGACGCCCACGCAGGTCAAGATCTGGTTCCAGAACCACCGCTACAAAATGAAGCGCCAGGCCAAGGACAAGGCGGCGCAGCAGCAACTGCAGCAGGACAGCGGCGGCGGCGGGGGCGGCGGGGGCACCGGGTGCCCGCAGCAGCAACAGGCTCAGCAGCAGTCGCCGCGACGCGTGGCGGTGCCGGTCCTGGTGAAAGACGGCAAACCGTGCCAGGCGGGTGCCCCCGCGCCGGGCGCCGCCAGCCTACAAGGCCACGCGCAGCAGCAGGCGCAGCACCAGGCGCAGGCCGCGCAGGCGGCGGCAGCGGCCATCTCCGTGGGCAGCGGTGGCGCCGGCCTTGGCGCACACCCGGGCCACCAGCCAGGCAGCGCAGGCCAGTCTCCGGACCTGGCGCACCACGCCGCCAGCCCCGCGGCGCTGCAGGGCCAGGTATCCAGCCTGTCCCACCTGAACTCCTCGGGCTCGGACTACGGCACCATGTCCTGCTCCACCTTGCTATACGGTCGGACCTGGTGA                                                      
Patient 3 c.530G>C p.(Gly177Ala)/ c.440G>C p.(Gly147Ala)ATGTGGTCCGGAGGCAGTGGGAAGGCGCGGGGCTGGGAGGCCGCGGCGGGAGGGAGGAGCAGCCCCGGCAGGCTCAGCCGCCGCCGAATCATGTCGATGAGTCCAAAGCACACGACTCCGTTCTCAGTGTCTGACATCTTGAGTCCCCTGGAGGAAAGCTACAAGAAAGTGGGCATGGAGGGCGGCGGCCTCGGGGCTCCGCTGGCGGCGTACAGGCAGGGCCAGGCGGCACCGCCAACAGCGGCCATGCAGCAGCACGCCGTGGGGCACCACGGCGCCGTCACCGCCGCCTACCACATGACGGCGGCGGGGGTGCCCCAGCTCTCGCACTCCGCCGTGGGGGGCTACTGCAACGGCAACCTGGGCAACATGAGCGAGCTGCCGCCGTACCAGGACACCATGAGGAACAGCGCCTCTGGCCCCGGATGGTACGGCGCCAACCCAGACCCGCGCTTCCCCGCCATCTCCCGCTTCATGGGCCCGGCGAGCGGCATGAACATGAGCGGCATGGGCGGCCTGGGCTCGCTGGGGGACGTGAGCAAGAACATGGCCCCGCTGCCAAGCGCGCCGCGCAGGAAGCGCCGGGTGCTCTTCTCGCAGGCGCAGGTGTACGAGCTGGAGCGACGCTTCAAGCAACAGAAGTACCTGTCGGCGCCGGAGCGCGAGCACCTGGCCAGCATGATCCACCTGACGCCCACGCAGGTCAAGATCTGGTTCCAGAACCACCGCTACAAAATGAAGCGCCAGGCCAAGGACAAGGCGGCGCAGCAGCAACTGCAGCAGGACAGCGGCGGCGGCGGGGGCGGCGGGGGCACCGGGTGCCCGCAGCAGCAACAGGCTCAGCAGCAGTCGCCGCGACGCGTGGCGGTGCCGGTCCTGGTGAAAGACGGCAAACCGTGCCAGGCGGGTGCCCCCGCGCCGGGCGCCGCCAGCCTACAAGGCCACGCGCAGCAGCAGGCGCAGCACCAGGCGCAGGCCGCGCAGGCGGCGGCAGCGGCCATCTCCGTGGGCAGCGGTGGCGCCGGCCTTGGCGCACACCCGGGCCACCAGCCAGGCAGCGCAGGCCAGTCTCCGGACCTGGCGCACCACGCCGCCAGCCCCGCGGCGCTGCAGGGCCAGGTATCCAGCCTGTCCCACCTGAACTCCTCGGGCTCGGACTACGGCACCATGTCCTGCTCCACCTTGCTATACGGTCGGACCTGGTGA                                                      
