# Supplementary material for: Deciphering the pathogenicity of three NKX2-1 variants in ultra-severe forms of childhood interstitial lung disease
Source: PLoS One. 2025 Dec 19;20(12):e0338446. doi: 10.1371/journal.pone.0338446 (PMC12716760; doi:10.1371/journal.pone.0338446)
Supplement: Attachment — Submitted filename: Soreze_PONE-D-25-14519_Point by point response.docx [file pone.0338446.s009.docx]

Journal Requirements:

When submitting your revision, we need you to address these additional requirements.

1. Please ensure that your manuscript meets PLOS ONE's style requirements, including those for file naming. The PLOS ONE style templates can be found at

https://journals.plos.org/plosone/s/file?id=wjVg/PLOSOne_formatting_sample_main_body.pdf and

<https://journals.plos.org/plosone/s/file?id=ba62/PLOSOne_formatting_sample_title_authors_affiliations.pdf>

2. Thank you for stating the following in the Competing Interests section:
“NN:

Grants or contracts from any entity

2024: CORTICONEHI: Clinical trial: Efficacy of methylprednisolone pulses in

neuroendocrine cells hyperplasia of infancy: an early phase study.

2023: Million Dollar Bike Ride project for Neuroendocrine Cell Hyperplasia of Infancy:

Genetic basis of neuroendocrine cell hyperplasia of infancy

2022: Chancellerie des Universités: Legs Poix, Molecular and phenotypic

characterization of interstitial lung disease n°2022000594

2022: RespiFIL grant for the development of an e-learning module for CT-scan in

childhood interstitial lung diseases (15 000 €)

2022: RespiFIL grant for the development of an online platform for the collection of

quality of life and transition questionnaires in rare lung disease (15 000 €)

Payment or honoraria for lectures, presentations, speakers bureaus, manuscript writing

or educational events

2022: La lettre du Pneumologue

Support for attending meetings and/or travel: 2023: ERS Travel grant

Leadership or fiduciary role in other board, society, committee or advocacy group, paid

or unpaid

2021-2025 Head of the ERS Clinical research collaboration for childhood ILD (CRC[1]chILDEU)

2017-2023 Treasurer of the Société française de pédiatrie (SFP)

2023- Treasurer and Scientific committee of the Société de Pneumologie Pédiatrique

et d’Allergologie (SP2A)

2023- Scientific and Scientific committee of the Société de Pneumologie de Langue

Française (SPLF)

AC:

All support for the present manuscript

Supply of vectors”

Please confirm that this does not alter your adherence to all PLOS ONE policies on sharing data and materials, by including the following statement: "This does not alter our adherence to PLOS ONE policies on sharing data and materials.” (as detailed online in our guide for authors http://journals.plos.org/plosone/s/competing-interests). If there are restrictions on sharing of data and/or materials, please state these. Please note that we cannot proceed with consideration of your article until this information has been declared.

Please include your updated Competing Interests statement in your cover letter; we will change the online submission form on your behalf.”

3. We note that your Data Availability Statement is currently as follows: All relevant data are within the manuscript and its Supporting Information files.

Please confirm at this time whether or not your submission contains all raw data required to replicate the results of your study. Authors must share the “minimal data set” for their submission. PLOS defines the minimal data set to consist of the data required to replicate all study findings reported in the article, as well as related metadata and methods (https://journals.plos.org/plosone/s/data-availability#loc-minimal-data-set-definition).

For example, authors should submit the following data:

- The values behind the means, standard deviations and other measures reported;
- The values used to build graphs;
- The points extracted from images for analysis.

All these data are submitted in the manuscript.

Authors do not need to submit their entire data set if only a portion of the data was used in the reported study.

If your submission does not contain these data, please either upload them as Supporting Information files or deposit them to a stable, public repository and provide us with the relevant URLs, DOIs, or accession numbers. For a list of recommended repositories, please see <https://journals.plos.org/plosone/s/recommended-repositories>.

All data are available on request to the authors.

If there are ethical or legal restrictions on sharing a de-identified data set, please explain them in detail (e.g., data contain potentially sensitive information, data are owned by a third-party organization, etc.) and who has imposed them (e.g., an ethics committee). Please also provide contact information for a data access committee, ethics committee, or other institutional body to which data requests may be sent. If data are owned by a third party, please indicate how others may request data access.

4. When completing the data availability statement of the submission form, you indicated that you will make your data available on acceptance. We strongly recommend all authors decide on a data sharing plan before acceptance, as the process can be lengthy and hold up publication timelines. Please note that, though access restrictions are acceptable now, your entire data will need to be made freely accessible if your manuscript is accepted for publication. This policy applies to all data except where public deposition would breach compliance with the protocol approved by your research ethics board. If you are unable to adhere to our open data policy, please kindly revise your statement to explain your reasoning and we will seek the editor's input on an exemption. Please be assured that, once you have provided your new statement, the assessment of your exemption will not hold up the peer review process.

5. PLOS ONE now requires that authors provide the original uncropped and unadjusted images underlying all blot or gel results reported in a submission’s figures or Supporting Information files. This policy and the journal’s other requirements for blot/gel reporting and figure preparation are described in detail at https://journals.plos.org/plosone/s/figures#loc-blot-and-gel-reporting-requirements and https://journals.plos.org/plosone/s/figures#loc-preparing-figures-from-image-files. When you submit your revised manuscript, please ensure that your figures adhere fully to these guidelines and provide the original underlying images for all blot or gel data reported in your submission. See the following link for instructions on providing the original image data: https://journals.plos.org/plosone/s/figures#loc-original-images-for-blots-and-gels.
In your cover letter, please note whether your blot/gel image data are in Supporting Information or posted at a public data repository, provide the repository URL if relevant, and provide specific details as to which raw blot/gel images, if any, are not available. Email us at plosone@plos.org if you have any questions.

6. Your ethics statement should only appear in the Methods section of your manuscript. If your ethics statement is written in any section besides the Methods, please move it to the Methods section and delete it from any other section. Please ensure that your ethics statement is included in your manuscript, as the ethics statement entered into the online submission form will not be published alongside your manuscript.

7. If the reviewer comments include a recommendation to cite specific previously published works, please review and evaluate these publications to determine whether they are relevant and should be cited. There is no requirement to cite these works unless the editor has indicated otherwise. 


[Note: HTML markup is below. Please do not edit.]

Reviewers' comments:

Reviewer's Responses to Questions

5. Review Comments to the Author

Please use the space provided to explain your answers to the questions above. You may also include additional comments for the author, including concerns about dual publication, research ethics, or publication ethics. (Please upload your review as an attachment if it exceeds 20,000 characters)

Reviewer #1: The authors present 3 cases of "ultra-severe forms of childhood interstitial lung disease" and a functional analysis of 3 NKX2.1 variants identified in the subjects. Actually, only one variant (#1) is novel and meets the aims in the title. The phenotype-genotype correlation is unclear for cases #2 and 3. The variants’ functional testing is not novel.
There are many grammatical issues and the language should be reviewed by a native English speaker.

**Major concerns:**

**Case reports
Case 1**
- Clinical presentation seems typical of Persistent pulmonary hypertension of the newborn (PPHN). The chest CT is of poor quality and cannot support a diagnosis of interstitial lung disease but rather shows diffusely decreased aeration.

We thank the reviewer for this comment. Chest CT of Patient 1 was performed while the patient was under ECMO support, what explains its poor quality. The CT chest was reviewed with an expert pediatric radiologist, and diffuse ground glass opacities (GGO) were attested. Chest Xray being generally less impacted by the presence of the cannulas than CT, we added in the Supplemental Materials a chest X-ray of Patient 1 showing GGO as well. This info was added lines 106-108 and in **Figure 1A**’s legend.

- The correct histological phenotype is acinar dysplasia, which is a developmental lung disease, not ILD.

We thank the reviewer for this comment. Acinar dysplasia is indeed a diffuse developmental lung disease. However, because it can manifest as an ILD pattern, with GGO, as in Patient 1, this disease is included in most childhood Interstitial lung diseases (chILD), as in Griese M. et al, *Eur Respir Rev*, 2018 (Groupe A1). We added this info in the discussion section lines 235-237.

- What was the purpose of surfactant protein immunostaining, and what is the relevance of the results in the context of the paper? In figure 1D legend, the authors report weak SP-B staining, which seems the opposite on the corresponding image. Considerations on protein expression in lung tissue should be supported by Western blot.

We thank the reviewer for this comment and this question. Surfactant protein immunostaining was performed to assess the function of NKX2-1 in patient lung biopsies, since NKX2-1 is a transcription factor of surfactant proteins. The weak staining of SP-B may indeed be difficult to observe due to the hyperplasia of type 2 alveolar cells. Although more cells are stained, they appear weaker than the cells of the control. This information has been added to **Figure 1**’s legend. Unfortunately, due to technical issues, we were not able to perform western blot in lung tissue from patients. This was added to the manuscript lines 113-116.

- How was genetic testing done? Which genes were tested? Was the variant inherited or de novo? Was a molecular caryotype performed (as TTF-1 deficiency can be caused by CNVs)?

We thank the reviewer for these questions. Genomic DNA was extracted from peripheral blood leukocytes of the probands and their parents. Sanger sequencing targeting NKX2-1 was performed for patients 1 and 2. It was performed on an ABI 3730XL automated capillary DNA sequencer (Applied Biosystems). Sequencing data were then analyzed through the Seqscape software.

In the meantime, NGS for the following PHT-related genes was also performed: *ACVRL1, BMP10, BMPR2, CAV1, EIF2AK4, ENG, EPHB4, FOXF1, GDF2, GLMN, KCNK3, KRIT1, PTEN, RASA1, SMAD4, SMAD9, SOX17, TBX4, TEK* (<http://www.cgmc-psl.fr/spip.php?article45>). This was added in the manuscript, in the methods section, lines 299-314.

As reported in PMID: 38035569 (doi: 10.1159/000534076), the variant was not found in the mother. The father was asymptomatic but did not consent to the DNA analysis. This was added in **Table 1**.

No karyotype or SNP arrays were performed in the three patients. CNVs can be detected using NGS targeted sequencing. For Patients 1 and 2, whose phenotypes were highly suggestive of brain-lung-thyroid syndrome, Sanger sequencing identified likely pathogenic variants, and confirmed the diagnosis. The heterozygosity of the variants confirmed the presence of two alleles, ruling out the possibility of a large deletion affecting the entire *NKX2-1* gene. For Patient 3, who carried a variant of uncertain significance, no deletion of the *NKX2-1* gene was identified by NGS. This was added in the manuscript, line 155-156.

- Was the thyroid gland absent, ectopic, or dysplastic?

We thank the reviewer for this question. The thyroid gland was present and in normal position. This information was added in the manuscript lines 107-108. We added in the **Supplemental data** some thyroid imaging of the Patient 1.

- Was there a neurological phenotype?

We thank the reviewer for this question. As reported in PMID: 38035569 (doi: 10.1159/000534076), the patient presented at birth with severe hypotonia and respiratory failure. However, it was impossible to discriminate if this hypotonia was related to her respiratory condition or to *NKX2-1* disease. This was added in the manuscript line and line 109-110, 231-235 (discussion and results + **Table 1**).

**Case 2**
- The clinical history is difficult to follow. When was the chest CT performed in the course? Was it interpreted as post-RSV changes, VILI, or ILD, or a combination? Genetics: same questions as above.

We thank the reviewer for these questions. Questions related to molecular aspects for patient 2 were answered ahead.

The patient was unknown from our hospital before the age of 2 years old when he presented with severe respiratory distress in a context of RSV infection. Because of the uncommon evolution and clinical signs of respiratory insufficiency such as digital clubbing and poor weight gain, a chest CT scan was performed leading to the diagnosis of ILD. Thus, this CT scan was interpreted as ILD, not post RSV changes nor VILI. Thereafter, congenital hypothyroidism was diagnosed and NGS analysis identified the de novo missense heterozygous *NKX2-1* variant c.493C>T p.(Arg165Trp).

The patient had congenital hypothyroidism. The thyroid gland was present and in normal position. We added in the supplemental data some thyroid imaging of patient 2. The patient had a slight hypotonia and neurodevelopmental delay but no chorea. The patient history was completed in the manuscript line 130-136

**Case 3**
- The clinical history is unclear. It is compatible with ILD. The chest CT seems indicative of PAH, not typical of ILD. Please provide a more accurate description. Same question as above for thyroid and brain.

We thank the reviewer for these comments. Patient 3 was not born in France, so the beginning of her medical history was difficult to retrieve. She presented with symptoms of respiratory failure at the age of 6 months old. Even though there were patterns of ILD on her chest CT scan, her medical history and lung biopsy were compatible with pulmonary hypertension. She didn’t have hypothyroidism and had no neurological impairment. We clarified the clinical history in the manuscript lines 143-150.

- The relevance of this case is questionable as molecular diagnosis remains unknown. Again, which genetic tests were performed?

We thank the reviewer for these questions. For patient 3, NGS targeting all the coding exons and the intronic flanking regions of the following list of genes was performed: *ABCA3, COPA, CSF2RA, CSF2RB, FARSA, FARSB, FLNA, GATA2, MARS1, NKX2-1, OAS1, SFTPA1, SFTPA2, SFTPB, SFTPC, STING1, and TBX4*. NGS was performed using KAPA Library Preparation and KAPA HyperCap Target Enrichment Probes (Roche Sequencing), and using a Miseq (Illumina) platform according to the manufacturer’s instructions. Sequence data were analyzed by using an in-house bioinformatics pipeline. Sequence reads in fastq format were aligned to the reference human genome (hg19) with BWA and Bowtie 2. Variant calling was performed with GATK and VarScan with a threshold for the variant allele fraction (VAF) of 10%. Variant calls in VCF format were then annotated through Annovar. As for Patients 1 and 2, we also performed NGS of pulmonary hypertension genes: *ACVRL1, BMP10, BMPR2, CAV1, EIF2AK4, ENG, EPHB4, FOXF1, GDF2, GLMN, KCNK3, KRIT1, PTEN, RASA1, SMAD4, SMAD9, SOX17, TBX4, TEK* (<http://www.cgmc-psl.fr/spip.php?article45>). This was added in the methods section of the manuscript lines 299-314.

NGS revealed a missense heterozygous variant in NKX2-1 c.440G>C p.(Gly147Ala). In the absence of another identified molecular cause for patient 3’s disease, we needed to ascertain (or to exclude) the role of the identified NKX2.1 variant. Finally, we demonstrated that her variant of NKX2.1 was not pathogenic and can be used as a molecular control in our different testing. This was added in the manuscript in the discussion lines 215-218.

- Functional analysis of the three variants. This section is more relevant and novel. However, the authors should explain the rationale for the tests performed. NKX2.1 deficiency mechanisms include defective nuclear translocation (Peca et al. Respiratory Research 2011, 12:115), haploinsufficiency (decreased transcription, decreased translation), or functional defects (lack of promoter transactivation in target genes) among others. This should be discussed in the manuscript. A table summarizing the findings would be welcome.

We thank the reviewer for these comments. We have clarified the rationale of the different tests performed in the manuscript in the methods section lines 330-331, lines 352-353, lines 360-361, and lines 372-373 and a new table was added (**Table 2**).

1/ Haploinsufficiency: we first performed Western Blot analysis to confirm proper expression of the different proteins despite the presence of these variants. Notably, we observed decreased expression of p.(Arg165Trp) and p.(Tyr214Cys), indicating possible haploinsufficiency.

2/ Functional defect: as we had identified three missense point variants in our patients, we hypothesized that they would be responsible for targeted promoter impairment. This hypothesis led us to conduct luciferase assays.

3/ Defective nuclear translocation: Furthermore, immunofluorescence assays were performed to ensure that the variants did not result in defective nuclear translocation, because 2 of 3 variants are located in the homeodomain of NKX2-1 which also contains the nuclear localization signal.

- On figure 3B, the control has poor and diffuse TF-1 staining, and doesn’t demonstrate nuclear staining, contrarily to the legend.

We thank the reviewer for this comment. In Figure 3B, the control referred to the empty vector and not to the control (wild type). We acknowledge that this was confusing and we modified the legend accordingly.

- For the Y214C variant, do your results suggest a combined mechanism of decreased expression (haploinsufficiency) and loss-of-function (impaired transactivation)? Again, a table comparing the different effects of the 3 variants would help.

As pointed out by the reviewer, we suspected that the p.(Tyr214Cys) variant was responsible for haploinsufficiency because of the results observed on western blots. We did not quantify the immunofluorescence staining. We also suspected loss-of-function and a dominant negative effect after observing impaired transactivation of the different promoters in the luciferase tests. The new **Table 2** is summarizing these points.

- R165W DNA-binding analysis is not novel (ref 14). The functional tests presented here are similar, and just confirm the published findings, i.e. domaicominant-positive effect in the lung, not in the thyroid. However, the authors report also partial haploinsufficiency.

We thank the reviewer for these comments. As underlined by the reviewer, our experiments confirmed the results of Guillot et al. This consistency was reassuring regarding the quality of our data and our ability to reproduce established results. Moreover, our western blot experiments indicated that this variant also leads to haploinsufficiency. This is now mentioned in the Discussion section lines 246-261.

How do you reconciliate these findings in order to explain the clinical impact?

We thank the reviewer for this question. Genotype-phenotype correlations are hard to affirm and the translation of the results of functional analyses to clinical findings are to assess with caution. However, our results suggest that the R165W variant is peculiarly severe since it has two different mechanisms of pathogenicity. We added this information in the discussion section lines 266-287.

Interestingly the dominant-positive effect is seen only on the SFTP-C promoter, not on SFTP-B. Can it depend on opposite effects on the promoter of SFTP-B or other surfactant-related proteins regulated by NKX2.1, such as ABCA3?

Thank you very much for this comment. We were intrigued to observe this differential effect on the two promoters SFTPB and SFTPC. We hypothesized that the R165W variant, located within the homeodomain, may impact transactivation in distinct ways. Notably, Stuart et al. *Communications Biology*, 2021, demonstrated that NKX2-1 possesses multiple binding sites with unequal functional roles. Furthermore, Minoo et al. *Molecular and Cellular Biology*, 2007, reported that NKX2-1 interacts with FOXA1 to regulate SP-C protein expression. Delestrain et al. *British Journal of Clinical Pharmacology*, 2021, found that some SFTPC variants lead to the accumulation of pro-SPC in the endoplasmic reticulum, thereby reducing mature SP-C levels.

Based on these findings, we proposed that increased promoter activation may result in the overproduction of specific isoforms, potentially causing pro-SPC accumulation in the endoplasmic reticulum. Thus, the unique activation profile of the SFTPC promoter observed in our study may be explained by these mechanisms.

These hypotheses were added in the discussion section lines 250-2261 with the cited references.

Minor concerns:
Typically, NKX2.1, SFTP-B, SFTP-C designate the gene, whereas TTF-1, SP-B, SP-C designates the protein. I suggest to adjust the whole text for clarity. Genes should be in italic.
Variants should be reported in the HGVS Nomenclature throughout the text; p.(Tyr214Cys) c.641A>G

We thank the reviewer for this comment. Both NKX2.1 and TTF1 are used for the protein, and mostly *NKX2.1* for the gene. We made the appropriated corrections and introduced TTF1 as well in the introduction lines 76-77.

Line 55-56: that are associated with ultra-severe forms of childhood interstitial lung disease (chILD) in infants: not a true statement

We thank the reviewer for this comment. We proceeded to the corrections in the manuscript lines 89-90.

Line 114: The patient was diagnosed with peripheral CH: when? Was he asymptomatic until age 2.5?

We thank the reviewer for these questions. It's unclear if the patient was previously asymptomatic. This is likely, as he had a neurodevelopmental delay. However, language and cultural barriers made communication with the family challenging. The paragraph was modified in the manuscript lines 128-133.

Line 115: architectural destruction with

We thank the reviewer for this comment. We proceeded to the correction in the manuscript line 133.

Line 123: weeks gestation

We thank the reviewer for this comment. We proceeded to the corrections in the manuscript lines 143-144.

Line 127-9: She developed PHT and required long-term supplemental oxygen therapy;
She died of refractory cardiac arrest… pulmonary vasodilators? died of right heart failure? These are not appropriate clinical descriptions.

We thank the reviewer for this comment. We proceeded to the corrections in the manuscript lines 147-150.

Line 198-204: this paragraph is not pertinent to the discussion

We thank the reviewer for this comment. We proceeded to the corrections in the manuscript lines 240-245. Corrections of this part of the discussion were also suggested by another reviewer.

Line 217-220: this paragraph is more background, not discussion

We thank the reviewer for this comment. We proceeded to the corrections in the manuscript line 93-96.

**Reviewer #2:**

This study by Yohan Soreze and colleagues investigates three NKX2-1 variants (Y214C, R165W, G147A) associated with severe childhood interstitial lung disease. Through functional assays, the authors demonstrate that Y214C is a loss-of-function mutation with no promoter activation, R165W shows mixed effects, and G147A appears benign. These findings support the pathogenicity of Y214C and R165W, with Y214C representing the most severe phenotype reported to date. Although the study focuses on only three variants and uses HEK293 cells, the authors enhance the physiological relevance by co-transfecting transcriptional partners.

The manuscript is generally well written and easy to follow. However, I have several questions and suggestions that I hope will help improve the clarity and scientific rigor of the work.

Major Comments
1. Statistics and Replicates
• Most assays appear to be performed in triplicates (n=3), which is insufficient to reliably assume normality or robustly estimate variance.

• Normality tests are not reported, and multiple comparisons are conducted without appropriate correction.
• Figure 4A and 4B for TG promoter seem to be the same plot, I am sure the authors placed it by mistake.

Recommendations:
• Increase the number of biological replicates to strengthen the statistical power; or
• If no additional experiments are planned, consider using non-parametric statistical tests (e.g., Kruskal–Wallis) with appropriate corrections for multiple comparisons (e.g., Dunn’s or FDR correction).

We thank the reviewer for these recommendations. Triplicate was a standard procedure that allowed us to obtain significant results. However, as requested to improve the statistical significance, we performed new replicates up to n=5 for of the most relevant analyses. Statistical analyses were remade using Kruskal-Wallis tests and post-hoc test for multiple pairwise comparison. Modifications were made in the results section line 171-182 and the **Figure 4A** was modified accordingly.

• Check Figure 4 plots for TG promoter and put the correct plots.
We thank the reviewer for this comment. It was obviously a mistake. Corrections were made in **Figure 4**.

2. Methodological Detail and Reproducibility
• Key details are missing from the Methods section, making replication difficult.

Recommendations:
• Clearly describe the reporter plasmids: specify promoter lengths, vector backbones, and whether they are commercially or publicly available (e.g., Addgene codes).
• If not available via repositories, consider uploading plasmid maps to the supplementary material.

We thank the reviewer for this comment. Plasmid maps were added to the **Supplementary data**.

• Consider including a known pathogenic or benign NKX2-1 variant as an internal control in the luciferase assays to contextualize the functional results.

We thank the reviewer for this suggestion. Including known pathogenic / benign variants as controls could indeed have been a smart approach. Since the R165W variant was already published and known to be pathogenic, it could be considered as a positive control of pathogenicity. On the other side, we eventually demonstrated that the G147A variant was a polymorphism, we consider it as a negative control for our experiments. We discussed this point in the discussion section of the manuscript line 212

3. Interpretation of R165W Functional Effects
• Please elaborate on the R165W variant. Where is it located within the NKX2-1 structure? Does the affected residue have any known structural or functional importance?

We thank the reviewer for this comment that was in common with Reviewer #1, The R165W variant is located in the homeodomain of NKX2.1 protein which is its functional domain and the site of DNA binding. Variants occurring in the homeodomain of NKX2.1 are likely to be responsible of DNA binding disruption. This info was added in the manuscript lines 288-291.

• The gain-of-function effect seen with only one promoter is very intriguing and a few extra experiments could be interesting.
Suggestion:
While luciferase assays are useful for assessing promoter activity, they are limited in their scope. Additional experiments, such as ChIP-qPCR or ChIP-seq at relevant promoter sites, could provide stronger mechanistic insight into the variant-specific effects. I understand this is a tedious technique, and this is just a suggestion for the future.

We thank the reviewer for this comment, Reviewer #1 also suggested extra experiments that we hope to be able to perform them in the future. This was added to the discussion section line 268-274.

4. Interpretation of Clinical Data
• The conclusion stating that “impairment of promoter transactivation led to acinar dysplasia…” may be overstated, given the current functional evidence.
Suggestion:
Rephrase the conclusion to reflect that while functional impairment correlates with clinical severity, a direct causal link has not been definitively established.

We thank the reviewer for this comment. We rephrased the conclusion section to better reflect the current evidences, with no overinterpretation of the data, lines 389-391.

Minor Comments

• Terminology: The term “next-generation sequencing (NGS)” is somewhat outdated. Please consider using “high-throughput sequencing” instead.

We thank the reviewer for this suggestion. Usually, “high-throughput sequencing” refers to whole exome or whole genome sequencing. Here, it is about gene panel sequencing and mid-throughput sequencing.

• Sequencing Methods: Clarify the type of sequencing used (e.g., exome, panel, genome). Describe how variants were called and filtered. Providing raw variant calling files as supplementary material would enhance transparency and allow reproducibility.

We thank the reviewer for this comment. Genomic DNA was extracted from peripheral blood leukocytes of the probands and their parents. Sanger sequencing targeting of NKX2-1 was performed for the patients 1 and 2. It was performed on an ABI 3730XL automated capillary DNA sequencer (Applied Biosystems). Sequencing data were then analyzed through the Seqscape software. For patient 3, next generation sequencing (NGS) targeting all the coding exons and the intronic flanking regions of the following list of genes was performed: *ABCA3, COPA, CSF2RA, CSF2RB, FARSA, FARSB, FLNA, GATA2, MARS1, NKX2-1, OAS1, SFTPA1, SFTPA2, SFTPB, SFTPC, STING1, and TBX4*. NGS was performed using KAPA Library Preparation and KAPA HyperCap Target Enrichment Probes (Roche Sequencing), and using a Miseq (Illumina) platform according to the manufacturer’s instructions. Sequence data were analyzed by using an in-house bioinformatics pipeline. Sequence reads in fastq format were aligned to the reference human genome (hg19) with BWA and Bowtie2. Variant calling was performed with GATK and VarScan with a threshold for the variant allele fraction (VAF) of 10%. Variant calls in VCF format were then annotated through Annovar. This was added in the methods section of the manuscript lines 299-314 and NGS raw results were added in the Supplemental data.

• Variant Nomenclature: Please follow HGVS recommendations. Use three-letter amino acid codes where appropriate (e.g., p.Tyr214Cys instead of Y214C). Tools like Mutalyzer can help the authors with this.

We thank the reviewer for this comment. We proceeded to the corrections all over the manuscript.

• Line 200 – Acinar Dysplasia Associations: The association between acinar dysplasia and mutations in TBX4, FGF10, and FGFR2 is now well supported across multiple studies. While molecular mechanisms remain incompletely understood, newer model systems (e.g., lung organoids) are likely to help us understand these pathways in the context of AD.

We thank the reviewer for this comment. We rephrased this section to modulate our statements lines 240-245.

• Align well Figure 4 dashes.

We thank the reviewer for noticing. We proceeded to the corrections in **Figure 4**.

**Reviewer #3:**

This article describes the pathogenicity of three variants of the NKX2-1 gene associated with lethal forms of childhood interstitial lung disease : two likely pathogenic and located in the homeodomain of the NKX2-1 protein (p.Tyr214Cys and p.Arg165Trp) and one likely benign (p.Gly147Ala).
The authors report clinical phenotypes, chest CT findings and lung histology (when available). Functional analyses of NKX2-1 variants were performed in HEK293T cells, assessing protein expression, subcellular localization and transactivation of lung and thyroid specific promoters with or without PAX8 cotransfection. Two patients carrying Y214C and R165W variants exhibited brain-lung-thyroid syndrome and severe interstitial lung disease, with one showing atypical acinar dysplasia. In contrast, the patient with the G147A variant presented isolated lung disease, primarily characterized by pulmonary hypertension. In vitro functional studies confirmed the pathogenicity of two variants, Y214C and R165W, while the G147A variant showed no deleterious effect. Western-blot analysis demonstrated reduced protein level for Y214C and R165W with no change in their cellular localization. The R165W variant increased transactivation of the SFTPC promoter, caused a non significant decrease in the SFTPB promoter activity, and significantly reduced transactivation of the TG and TPO promoters, even with PAX 8 cotransfection. The Y214C variant significantly impaired transactivation of all tested promoters.
Surfactant disorders caused by NKX2-1 mutations are responsible for rare lung disease. To date, few functional studies have assessed the impact of NKX2-1 variants, making this manuscript particularly valuable for correlating genotype and phenotype. The methods used are appropriate to investigate the functional consequences of these variants.

However, the manuscript requires some minors revisions :
• Page 4, ligne 87 : « Heterozygous pathogenic variants of NKX2-1 are associated with brain-lung-thyroid syndrome » : 56% of patients carrying NKX2-1 mutations present with brain-lung-thyroid syndrome while the others exhibit mono or bi-organ involvement, as observed in patient 3 described in this study. It would therefore be relevant to specify this point in the manuscript.

We thank the reviewer for this comment. We proceeded to the corrections in the manuscript lines 89-90.

• Page 6 line 114 : « The patient was diagnosed with peripheral CH » The abbreviations « CH » should be defined when first mentionned in the manuscript.

We thank the reviewer for noticing. CH refers to Congenital Hypothyroidism. We now have defined the abbreviation at first occurrence in line 91.

• Page 6 line 142 : There appears to be typographical error in the variant name « Y214C », where the « C » (for cysteine) is missing.

We thank the reviewer for noticing. We proceeded to the corrections in the manuscript lines 164-166.

• Page 6 line 142 : « Western blot analysis showed that
R165W and Y214 expression levels were significantly lower than those in WT and G147A » Although the mutated NKX2-1 protein shows reduced expression in HEK293 cells compared to the WT, NKX2-1 expression was reported as normal in the lung biopsy of patient 1. The authors should discuss this apparent discrepancy.

We thank the reviewer for this relevant comment. Indeed, immunohistochemistry for patient 1 appears to show normal NKX2.1 staining. This observation could be explained by several mechanisms.

- The disease being dominant, the patients have one normal allele and one mutated allele. Thus, WBs using reflect the expression of the mutated allele (mono-transfection of the mutant cDNA) whereas the IHC performed on the patient’s lung biopsy reflects the global expression of NKX2.1, including the expression of the normal allele which can make it difficult to observe a decreased staining.

- This observation could also be related to hyperplasia of alveolar epithelial cells seen in acinar dysplasia. The cells are not actual type 2 alveolar epithelial cells, so the staining has to be interpreted cautiously. The lung biopsy of patient 2 (p.(Arg165Trp)) would have been very interesting to see if the decreased NKX2-1 staining reported by Guillot L, et al. 2009 was present in our patient as well.

We added these statements in the discussion lines 219-226.

• The nomenclature used to describe the protein variants is inconsistent throughout the manuscript. The authors should standardize the naming convention to ensure clarity and coherence. For example, the same variant is referred to as NKX2-1_p.Y214C (page 7 line 157), NKX2-1_Y214C (page 7 line 162), Y214C (page 7 line 145) or p.Tyr214Cys (Page 5 line 106).

We thank the reviewer for this comment. We proceeded to the corrections all over the manuscript.

• Figure 3 : The authors should standardize the legends between the western-blot and Immunofluorescence panels to facilitate interpretation. For example, does « Empty vector » in the western-blot correspond to « Control » in the immunofluorescence ? Does « NKX2-1 » in the immunofluorescence refer to the « WT » condition in the western-blot ? In addition, the nomenclature used to describe the protein variants in the figure should be consistent with that used in the main text.

We thank the reviewer for this comment. We clarified the legends: “control” is the “empty vector” and “NKX2-1” is the wild type. We proceeded to the corrections in the manuscript line and in the **Figures** line 170.

• In Page 9, line 201 : The statement « Our study contributes to the involvement of NKX2-1 in lung development, endorsing an important role between the pseudoglandular (8WG) and canalicular stages (26 WG) » would benefit from further support. The authors are encouraged to provide additional data, references, or justification to substantiate this claim.

We thank the reviewer for this comment. This statement was rephrased and modulated as follow. In Maquet et al. *J Clin Endocrinol Metab*. 2009, and Soreze et al. *Neonatology*. 2024, articles, the early expression of NKX2.1 in the lung was demonstrated NKX2-1 as well as its implication in the branching of the lobar bronchi and in the later stages of lung development. Our study sought to extend the understanding of the brain-lung-thyroid syndrome phenotype by including diffuse developmental disorders of the lung, thereby prompting inquiry into the mechanistic effect of NKX2-1 on pulmonary development. This was added in the manuscript lines 243-248.

• In page 9, line 208 : « There was a significant increase in the transactivation of SFTPC promoter, confirming a dominant positive effect that was previously suggested » The authors demonstrate that the R165W variant enhances transactivation of the SFTPC promoter. However, the potential pathogenic mechanism underlying this effect remains unexplored. They should discuss how increased SFTPC expression possibly through ER stress, disrupted alveolar type II cell homeostasis or inflammatory signaling might contribute to the development of interstitial lung disease.

We thank the reviewer for this comment. This point was also rose by another reviewer. Delestrain et al. *British Journal of Clinical Pharmacology,* 2021, found that certain *SFTPC* variants lead to the accumulation of pro-SPC in the endoplasmic reticulum, thereby reducing mature SP-C levels. Based on these findings, we proposed that increased promoter activation may result in an overproduction of specific isoforms, potentially causing pro-SPC accumulation in the endoplasmic reticulum and thus increased ER stress. This could explain the phenotype observed in our patient with the over activation of *SFTPC* promoter. Furthermore, Minoo et al. *Molecular and Cellular Biology*, 2007, reported that NKX2-1 interacts with FOXA1 to regulate SP-C protein expression. This can also be part of the mechanisms. This was emphasized in the Discussion section lines 253-264.

• In Page 10, line 225 : « The two pathogenic variants reported here, Y214C and R165W, are single nucleotide substitutions resulting in missense variants in the homeodomain of NKX2-1 (DNA-binding site). This binding impairment might explain the highly deleterious consequences on protein function and could be used as a predictive factor of severity ». The authors should reconsider the phrasing regarding the genotype-phenotype association, as this correlation is not consistently observed in all cases. Notably, whole gene deletions have been reported to cause milder disease phenotypes. It would be helpful to clarify whether the severity of the disease is influenced solely by the type of mutation or also by its location within the gene. Furthermore, the authors are encouraged to discuss additional factors that may contribute to the variability in disease severity, such as modifier genes or epigenetic mechanisms.

We thank the reviewer for this comment. We modified this section in the discussion and argued more with supplemental references, lines 278-294

To date, genotype-phenotype correlations for NKX2-1 variants are challenging given the variety of molecular variations and clinical presentations. Hamvas et al. *Chest*. 2013. reported patients with large deletions encompassing NKX2-1 and adjacent genes and manifesting with an early-onset complete brain-lung-thyroid syndrome. Conversely, patients with whole-gene deletions exhibited milder pulmonary disease. The authors further hypothesized that certain variants may disrupt the interaction of NKX2.1 with its molecular partners, and that epigenetic factor could contribute to phenotypic variability.

Another example is in the study of Thorwarth et al. *J Med Genet*. 2014, where the authors suggested that a single amino acid substitution within the homeodomain of NKX2-1 was less tolerated than other molecular variations. More recently, Michel et al. *Front Med*. 2025, conducted a systematic review including 38 studies and 148 patients. Their findings highlighted a broad spectrum of symptoms and genetic variations, and pointed out that the three out of 115 patients whose disease was complicated by a lung had nonsense variants.
